# Severe Serotonin Syndrome With Acute Respiratory Failure Following Ayahuasca and Dextromethorphan Use: A Case Report

**DOI:** 10.7759/cureus.114211

**Published:** 2026-08-09

**Authors:** Sean Pack, Tyler R Ellett, Jamie Pham, Yahya Ahmad

**Affiliations:** 1 Internal Medicine, St. Francis - Emory Healthcare, Columbus, USA; 2 Critical Care Medicine, St. Francis - Emory Healthcare, Columbus, USA

**Keywords:** acute respiratory failure, ayahuasca, dextromethorphan, monoamine oxidase inhibitors, serotonin syndrome

## Abstract

Ayahuasca is a psychoactive botanical brew originating from Amazonian ceremonial traditions, containing monoamine oxidase inhibitors (MAOIs) derived from *Banisteriopsis caapi* and N,N-dimethyltryptamine from *Psychotria viridis* or *Diplopterys cabrerana*. Its use has expanded globally for perceived antidepressant and anti-addictive effects. However, the MAOI content of ayahuasca predisposes users to serotonergic toxicity when combined with other serotonergic agents. We present the case of a 46-year-old man who developed severe serotonin syndrome (SS) complicated by acute respiratory failure after ingesting ayahuasca over three days, followed by over-the-counter dextromethorphan (DXM) and diphenhydramine. He required emergent intubation and intensive care unit (ICU) admission. Management with intravenous benzodiazepines, oral cyproheptadine, and supportive care led to rapid clinical improvement, with extubation on ICU day 2 and discharge on hospital day 3. Severe SS attributable to ayahuasca is rarely documented, and to our knowledge this is the first report of ayahuasca combined with over-the-counter DXM and diphenhydramine producing SS severe enough to cause acute respiratory failure. This case highlights the life-threatening potential of combining ayahuasca with commonly available serotonergic medications and underscores the need for clinician awareness of these interactions.

## Introduction

Serotonin syndrome (SS) is a potentially life-threatening hypermetabolic condition resulting from excessive serotonergic activity in the central nervous system [1]. It is characterized by a clinical triad of mental status changes, autonomic instability, and neuromuscular hyperactivity [1,2]. The syndrome most commonly occurs when two or more serotonergic agents are used concurrently, particularly when a drug that increases intrasynaptic serotonin is combined with a monoamine oxidase inhibitor (MAOI) [1,3].

Ayahuasca is a traditional Amazonian psychoactive brew prepared from Banisteriopsis caapi, which contains β-carboline alkaloids (harmine, harmaline, and tetrahydroharmine), and Psychotria viridis or Diplopterys cabrerana, which contain N,N-dimethyltryptamine (DMT) [4,5]. The β-carboline alkaloids function as potent reversible inhibitors of monoamine oxidase A (MAO-A), preventing the first-pass degradation of DMT and enabling its oral bioavailability [6,7]. DMT itself acts as an agonist at serotonin 5-HT1A, 5-HT2A, and 5-HT2C receptors, as well as a serotonin transporter and norepinephrine transporter inhibitor [5]. The global use of ayahuasca has expanded significantly in recent decades, driven by interest in its potential therapeutic effects for depression, anxiety, and substance use disorders [5,8].

Dextromethorphan (DXM), a widely available over-the-counter antitussive, inhibits serotonin reuptake and has been well-documented to cause SS when combined with MAOIs, even at therapeutic doses [9,10]. Diphenhydramine, a first-generation antihistamine, also possesses serotonin reuptake inhibitory properties and may contribute to serotonergic toxicity [11,12]. The combination of ayahuasca's MAOI activity and the serotonergic properties of DXM and diphenhydramine creates a pharmacologically high-risk scenario for severe SS.

Despite this well-characterized pharmacological plausibility, clinically documented cases are exceedingly rare. A systematic thematic review of ayahuasca and DMT adverse events found that serotonin toxicity attributed to ayahuasca has been described only in isolated reports involving concurrent prescription serotonergic psychiatric medications and identified no confirmed cases within the literature reviewed; the physical adverse events most commonly reported were mild and self-limited, such as nausea and vomiting [8]. Reviews of serotonin toxicity associated with serotonergic psychedelics likewise conclude that severe reactions are infrequently reported relative to the scale of contemporary ayahuasca use [3]. The published literature on DXM-associated SS, in contrast, derives predominantly from co-exposure with irreversible synthetic MAOIs such as phenelzine and isocarboxazid, and existing poison-center guidance addresses that pharmaceutical pairing rather than plant-derived reversible MAO-A inhibitors [9]. The clinical intersection of these two literature studies, a reversible plant-derived MAO-A inhibitor followed by an unremarkable over-the-counter cough and allergy preparation, remains largely undescribed, and the severity that such an exposure can reach has not been characterized.

We present a case of severe SS with acute respiratory failure in a patient who consumed ayahuasca and subsequently took over-the-counter medications containing DXM and diphenhydramine. To our knowledge, this is the first report of this exposure combination producing serotonergic toxicity severe enough to cause acute respiratory failure requiring emergent intubation and ICU admission. This case therefore extends a limited literature in which ayahuasca-associated serotonin toxicity has been considered rare, generally mild, and largely theoretical when non-prescription agents are involved, and it demonstrates that a widely used and easily obtained antitussive can precipitate a critical-illness presentation days after the last ayahuasca dose.

This article was previously presented as an abstract at the 2026 American Thoracic Society (ATS) Annual Meeting on May 18, 2026.

## Case presentation

A 46-year-old man with a past medical history significant for Factor V Leiden presented to the emergency department with agitation, hyperthermia, and psychomotor disturbance after reportedly ingesting an unknown substance. Further history revealed that the patient had consumed ayahuasca over the course of three days. Following his last ingestion, he took Mucinex DM (guaifenesin/dextromethorphan) and diphenhydramine for cough and allergy symptoms.

On arrival, vital signs were notable for a respiratory rate of 27 breaths per minute, blood pressure of 166/98 mmHg, oxygen saturation of 93% on room air, and temperature of 38.9°C (102.0°F). Physical examination revealed tachycardia, diaphoresis, coarse rhonchi bilaterally, and accessory muscle use. The patient was agitated with psychomotor disturbance.

**Figure 1:**
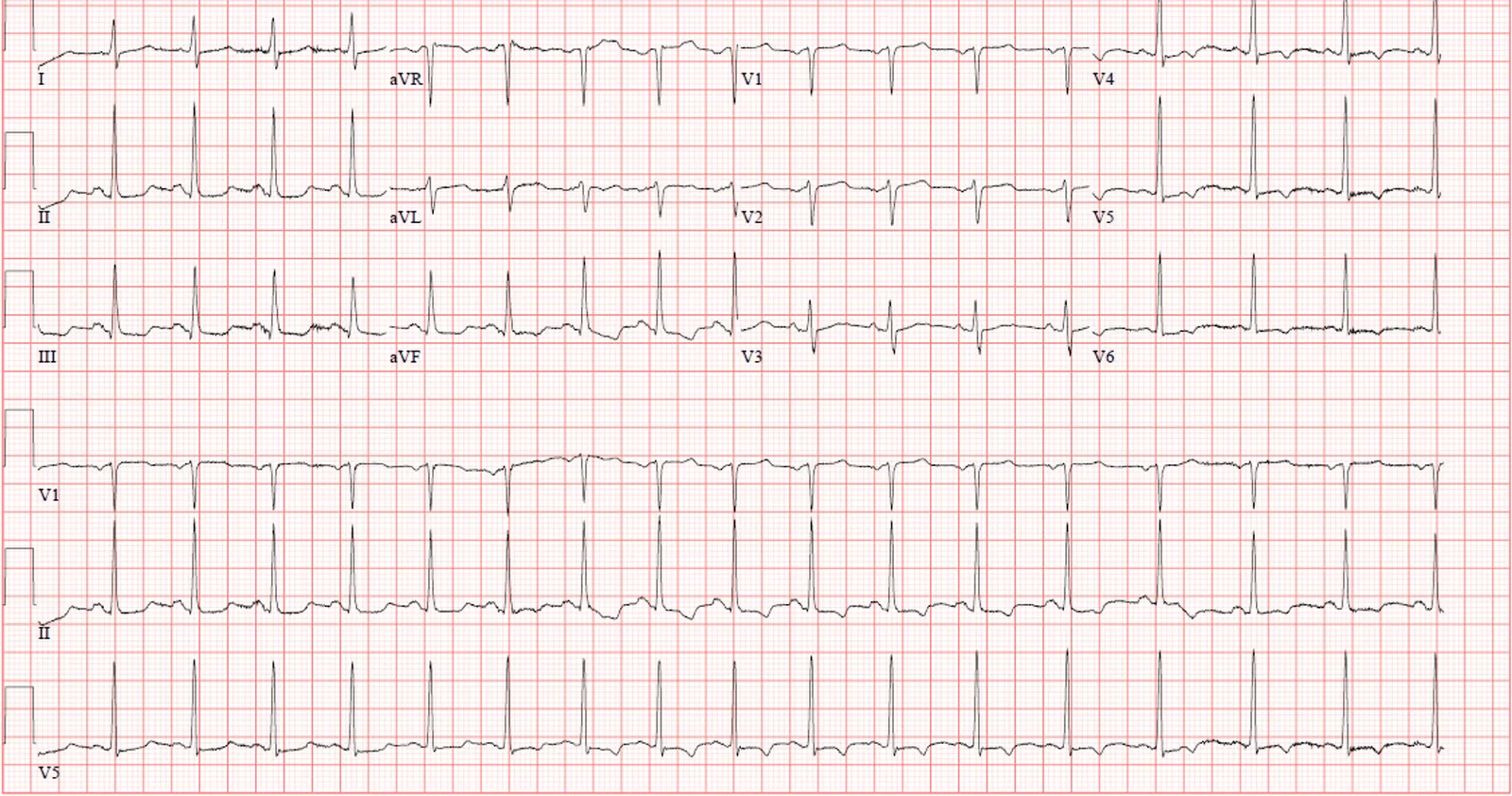
Initial Electrocardiogram Demonstrating Sinus Tachycardia with Inferolateral T-Wave Abnormalities

**Figure 2:**
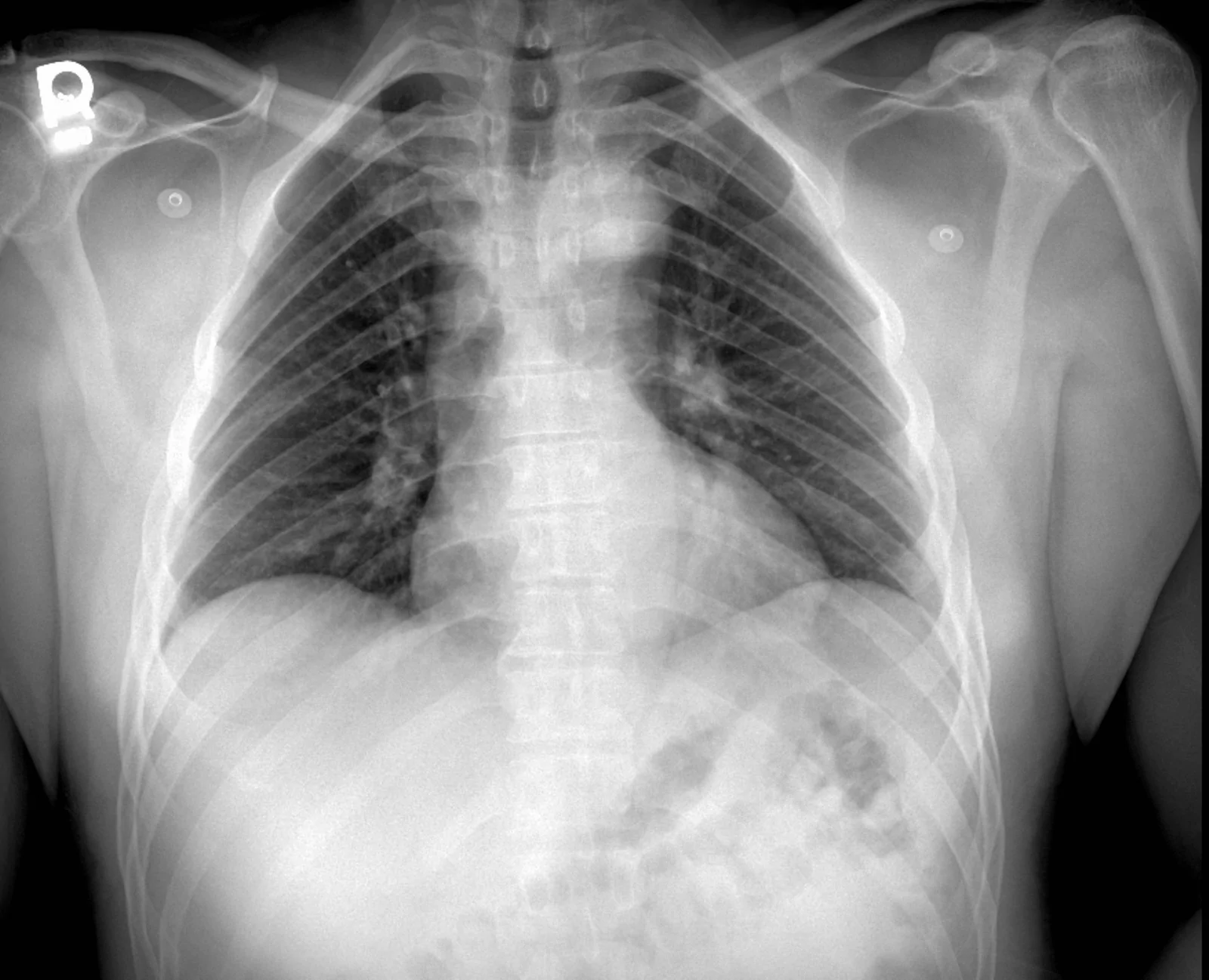
Chest Radiograph Demonstrating No Acute Cardiopulmonary Abnormalities

Laboratory evaluation demonstrated an elevated creatine kinase (CK) of 1,432 IU/L and aspartate aminotransferase (AST) of 47 IU/L, consistent with early rhabdomyolysis (Table 1). Urine toxicology screen was positive for benzodiazepines. Electrocardiogram (ECG) showed sinus tachycardia with inferolateral T-wave abnormalities (Figure 1). Chest imaging was unremarkable (Figure 2).

**Table 1:** Complete Blood Count (CBC), Comprehensive Metabolic Panel (CMP), and Additional Laboratory Findings on Admission

Laboratory Test	Result	Reference Range
Complete Blood Count (CBC)		
WBC	8.40 ×10³/µL	4.0-11.0 ×10³/µL
RBC	4.66 ×10⁶/µL	4.5-5.9 ×10⁶/µL
Hemoglobin (Hgb)	14.7 g/dL	13.5-17.5 g/dL
Hematocrit (Hct)	42.20%	41-53%
Mean Corpuscular Volume (MCV)	90.5 fL	80-100 fL
Mean Corpuscular Hemoglobin (MCH)	31.6 pg	27-33 pg
Mean Corpuscular Hemoglobin Concentration (MCHC)	34.9 g/dL	32-36 g/dL
Red Cell Distribution Width (RDW)	12.10%	11.5-14.5%
Platelets	237 ×10³/µL	150-400 ×10³/µL
Comprehensive Metabolic Panel (CMP) and Additional Labs		
Sodium	139 mmol/L	135-145 mmol/L
Potassium	3.7 mmol/L	3.5-5.0 mmol/L
Chloride	104 mmol/L	98-107 mmol/L
CO₂ (Bicarbonate)	25 mmol/L	22-29 mmol/L
Alkaline Phosphatase	64 U/L	44-147 U/L
AST	47 U/L (H)	10-40 U/L
ALT	35 U/L	7-56 U/L
BUN	21 mg/dL	7-20 mg/dL
Glucose	123 mg/dL	70-99 mg/dL
Creatinine	1.2 mg/dL	0.7-1.3 mg/dL
eGFR	76 mL/min/1.73m²	>60 mL/min/1.73m²
Calcium	9.0 mg/dL	8.5-10.5 mg/dL
Phosphorus	3.3 mg/dL	2.5-4.5 mg/dL
Total Protein	7.8 g/dL	6.0-8.3 g/dL
Albumin	4.8 g/dL	3.5-5.0 g/dL
Total Bilirubin	0.5 mg/dL	0.1-1.2 mg/dL
Anion Gap	10 mmol/L	8-16 mmol/L
Ammonia	32 µmol/L	15-45 µmol/L
Hemoglobin A1c	6.50%	<5.7%
Lactic Acid, Venous	1.0 mmol/L	0.5-2.2 mmol/L
Magnesium	2.1 mg/dL	1.7-2.2 mg/dL
Creatine Kinase (CK)	1432 U/L	30-200 U/L
Troponin, Baseline	4.6 ng/L	<14 ng/L
Troponin, 1-hour	5.1 ng/L	<14 ng/L

The patient's clinical course rapidly deteriorated, with the development of acute respiratory failure characterized by alternating apneic and tachypneic episodes. Emergent endotracheal intubation was performed, and the patient was admitted to the intensive care unit (ICU) with a clinical diagnosis of severe serotonin syndrome.

ICU management included intravenous isotonic saline for volume resuscitation, oral cyproheptadine (2 mg every two hours) administered via nasogastric tube as a 5-HT2A antagonist, and intravenous midazolam infusion (1 mg/hour) for sedation and control of neuromuscular rigidity. Over the ensuing 24 hours, the patient's hemodynamics stabilized, fever resolved, and ventilatory parameters normalized. He was successfully extubated on ICU day 2 and discharged from the hospital on day 3 in stable condition with no residual neurological deficits.

## Discussion

This case illustrates the life-threatening potential of combining ayahuasca with commonly available over-the-counter serotonergic medications, a combination that, to our knowledge, has not previously been reported to produce SS of this severity. The pharmacological basis for this interaction is well-established. Ayahuasca contains β-carboline alkaloids, primarily harmine, harmaline, and tetrahydroharmine, that act as potent reversible inhibitors of MAO-A [4,6,7]. MAO-A is the principal enzyme responsible for the metabolic degradation of serotonin in the central nervous system. When MAO-A is inhibited, the addition of agents that increase intrasynaptic serotonin through reuptake inhibition, such as DXM, produces synergistic serotonergic toxicity [3,9].

DXM is a sigma-1 receptor agonist and NMDA receptor antagonist that also inhibits serotonin reuptake [9]. SS has been reported with DXM even at therapeutic doses when combined with MAOIs, including phenelzine and isocarboxazid [9]. The American Association of Poison Control Centers guidelines specifically warn against the combination of DXM with MAOIs due to the risk of SS [9]. Diphenhydramine, while primarily an H1 antihistamine, has been shown to inhibit serotonin reuptake and may exacerbate serotonergic toxicity [11,12]. The FDA drug label for diphenhydramine notes that MAO inhibitors prolong and intensify its effects [13].

A critical aspect of this case is the temporal relationship between ayahuasca consumption and subsequent medication use. Although the β-carboline alkaloids in ayahuasca are reversible MAO-A inhibitors with relatively short half-lives compared to irreversible synthetic MAOIs, the patient consumed ayahuasca over three consecutive days, which may have resulted in sustained MAO-A inhibition [14]. The subsequent ingestion of DXM and diphenhydramine during this period of residual MAO inhibition likely precipitated the severe serotonergic crisis. This temporal pattern is clinically important because it indicates that the window of risk extends beyond the period of acute intoxication, when users are most likely to consider themselves no longer under the influence of the brew.

SS is a clinical diagnosis, established using validated decision rules rather than any confirmatory laboratory test. The two most widely applied are the Sternbach criteria and the Hunter Serotonin Toxicity Criteria [15,16]. In the setting of documented serotonergic exposure, this patient demonstrated mental status change with agitation, hyperthermia (38.9°C), diaphoresis, hypertonia, and autonomic instability manifested as hypertension and tachycardia, satisfying the Sternbach criteria for SS [16]. Formal application of the Hunter criteria was not possible in this case. The Hunter decision rules require the presence of clonus, spontaneous, inducible, or ocular, or the combination of tremor and hyperreflexia, in each instance in the setting of serotonergic agent exposure [15]. Clonus and deep tendon reflex assessment were not documented prior to emergent endotracheal intubation and initiation of sedation, a constraint frequently encountered in critically ill patients in whom airway management necessarily precedes detailed neurologic examination. The elevated CK of 1,432 IU/L supported the presence of significant neuromuscular hyperactivity and the early rhabdomyolysis characteristic of severe cases [1,10].

The differential diagnosis included neuroleptic malignant syndrome (NMS), anticholinergic toxicity, malignant hyperthermia, and sympathomimetic toxicity. NMS was considered unlikely given the absence of antipsychotic exposure and the rapid onset of symptoms. Anticholinergic toxicity was excluded by the presence of diaphoresis and hyperactive bowel sounds, which contrast with the dry, flushed skin and absent bowel sounds typical of anticholinergic poisoning [1]. The medication history and clinical trajectory were most consistent with SS.

Management of severe SS requires a multimodal approach including discontinuation of all serotonergic agents, aggressive supportive care, benzodiazepine sedation, and administration of 5-HT2A antagonists [1,17]. Cyproheptadine, a first-generation antihistamine with 5-HT2A antagonist properties, is the recommended pharmacologic therapy, although its efficacy has not been rigorously established in controlled trials [1,18,19]. Boyer and Shannon recommend an initial dose of 12 mg followed by 2 mg every two hours if symptoms persist, with maintenance dosing of 8 mg every six hours, noting that 12-32 mg over 24 hours binds 85-95% of serotonin receptors [1]. In this case, cyproheptadine was administered at 2 mg every two hours via nasogastric tube without the recommended 12 mg loading dose. This regimen was selected because the patient was concurrently receiving a continuous midazolam infusion that provided adequate control of agitation and neuromuscular rigidity, and the lower cyproheptadine dose was considered sufficient in that context. The cumulative 24-hour dose was therefore below the 12-32 mg range reported to achieve substantial serotonin receptor occupancy [1].

Benzodiazepines are essential in SS management regardless of severity, as they control agitation, reduce neuromuscular hyperactivity, and blunt the hyperadrenergic component of the syndrome [1,17]. The use of midazolam infusion in this case provided continuous sedation and rigidity control during mechanical ventilation. Physical restraints should be avoided, as they may worsen lactic acidosis and hyperthermia through enforced isometric muscle contractions [1].

The rapid clinical improvement observed in this patient, with hemodynamic stabilization and fever resolution within 24 hours, extubation on ICU day 2, and discharge on hospital day 3, is consistent with the expected course of SS when precipitating agents are discontinued and appropriate treatment is initiated [1,2]. The reversible nature of the β-carboline MAO inhibition, in contrast to irreversible synthetic MAOIs, likely contributed to the relatively rapid resolution [14].

This case has important public health implications. The global expansion of ayahuasca use, particularly in Western countries where it is consumed outside traditional ceremonial contexts, increases the likelihood of inadvertent drug interactions [8,20]. Users may not recognize that commonly available over-the-counter medications such as DXM-containing cough suppressants and diphenhydramine pose serious risks when combined with ayahuasca's MAOI activity. Fatalities have been reported with the combination of MAOIs and DXM [20]. Clinicians should be aware that patients presenting with unexplained agitation, hyperthermia, and neuromuscular hyperactivity may have consumed ayahuasca or similar plant-based preparations, and a thorough substance use history, including specific inquiry about plant-based psychedelic preparations, is essential.

This report describes a single patient, and the causal relationship between ayahuasca ingestion and the subsequent serotonergic crisis is inferred from temporal association and established pharmacology rather than definitively demonstrated. Quantitative toxicological confirmation was not obtained; serum and urine assays for harmine, harmaline, tetrahydroharmine, DMT, and dextromethorphan were not performed, and the alkaloid composition and quantity of the ingested preparation are unknown. Ayahuasca preparations vary substantially in alkaloid content. The doses and timing of DXM and diphenhydramine ingestion were obtained by patient report. As noted above, the absence of documented clonus and reflex examination precluded formal application of the Hunter Serotonin Toxicity Criteria. Finally, because benzodiazepines, cyproheptadine, and supportive care were administered concurrently, the clinical improvement cannot be attributed to any single intervention.

These limitations reflect broader gaps in the evidence base. Prospective toxicological characterization of ayahuasca preparations, pharmacokinetic studies defining the duration of clinically meaningful MAO-A inhibition following repeated dosing, and structured surveillance of adverse events associated with psychedelic use would substantially improve risk assessment for this increasingly common exposure.

## Conclusions

This case demonstrates that the combination of ayahuasca and over-the-counter serotonergic medications, specifically dextromethorphan and diphenhydramine, can precipitate severe SS with acute respiratory failure requiring emergent intubation and ICU admission, a presentation that, to our knowledge, has not previously been reported. Early recognition, prompt airway management, and treatment with benzodiazepines and cyproheptadine resulted in rapid clinical improvement and full recovery. As ayahuasca use continues to expand globally, clinicians must maintain a high index of suspicion for serotonergic toxicity in patients with recent psychedelic exposure and should counsel patients about the dangers of combining ayahuasca with serotonergic agents, including commonly available over-the-counter medications.
